# Androgenrezeptor-gerichtete Therapien – klinisch relevante Interaktionen vermeiden

**DOI:** 10.1055/a-2593-1605

**Published:** 2025-06-04

**Authors:** Hans-Peter Lipp, Gunhild von Amsberg, Axel S. Merseburger, Steffen Rausch, Tilman Schöning, Jörg Klier, Peter J. Goebell

**Affiliations:** 1 Lehrbeauftragter für Pharmaökonomie und -epidemiologie, Pharmazeutisches InstitutUniversität TübingenTübingenGermany; 2 Onkologisches Zentrum/ Martini-Klinik 37734 Universitätsklinikum Hamburg-EppendorfHamburgHamburgGermany; 3 UrologyUniversitätsklinikum Schleswig-Holstein, Campus LübeckLübeckGermany; 4 Klinik für UrologieUniversitätsklinik TübingenTübingenGermany; 5 Universitätsapotheke 27178 Universitätsklinikum HeidelbergHeidelbergBaden-WürttembergGermany; 6 UrologieUrologie Bayenthal in KölnKölnGermany; 7 Urologische und kinderurologische KlinikUniversitätsklinikum ErlangenErlangenGermany

**Keywords:** Prostatakarzinom, Arzneimittelinteraktionen, Polypharmazie, Pharmakokinetik, Cytochrom-P450-Enzymsystem, prostate cancer, drug interactions, polypharmacy, pharmacokinetics, cytochrome P450 enzyme system

## Abstract

Die Behandlung älterer Patienten erfordert eine besondere Aufmerksamkeit für Arzneimittelinteraktionen, da mit einer Zunahme der altersbedingten Morbidität eine Polypharmazie immer relevanter wird. Insbesondere bei Medikamenten mit einer geringen therapeutischen Breite können Arzneimittelinteraktionen klinische Konsequenzen haben, die eine Anpassung der Dosis oder das Beenden oder Wechseln der Begleitmedikation erfordern. Oft sind diese Interaktionen auf eine pharmakokinetische Veränderung des Arzneimittelabbaus über das Cytochrom-P450(CYP)-Enzymsystem zurückzuführen, vor allem über CYP3A4. Diese Enzymisoform ist an dem Metabolismus von etwa der Hälfte der therapeutisch verwendeten Arzneimittel beteiligt, sodass sich auch viele CYP3A4-Substrate unter den eingesetzten Medikamenten bei Prostatakrebspatienten befinden. Unter den starken CYP3A4-Induktoren Apalutamid und Enzalutamid sind in diesem Zusammenhang – im Gegensatz zu Darolutamid – in vielen Fällen deutlich erniedrigte Plasmakonzentrationen bei den gleichzeitig verabreichten Arzneimitteln zu erwarten. Bei Abirateron handelt es sich hingegen um einen moderaten CYP2D6-Inhibitor. Da über dieses Isoenzym deutlich weniger Wirkstoffe biotransformiert werden, ist das Interaktionsspektrum anders zu bewerten. Um im Vorfeld einer Pharmakotherapie das Ausmaß einer Wechselwirkung besser abschätzen zu können, ist es hilfreich, die Abbauwege der einzelnen Wirkstoffe besser zu verstehen, um im Zweifel rechtzeitig das Therapiemonitoring intensivieren, Dosisveränderungen und Wechsel einer Pharmakotherapie vornehmen zu können.

## Abstract (en)

Pharmacotherapy of older patients requires special attention to drug interactions, based on an increase in morbidity and elevated need for polypharmacy. Especially for drugs with a narrow therapeutic index, interactions may require dose adjustments, discontinuation or switching of co-medication. These interactions are frequently based on pharmacokinetic alterations of drug metabolism via the cytochrome P450 (CYP) enzyme system, mainly via CYP3A4. This isoform is involved in the metabolism of about half of therapeutically applied drugs, including many agents commonly used in patients with prostate cancer. As a consequence, an impressive decrease in plasma levels of coadministered drugs has to be expected during the use of the potent CYP3A4 inducers apalutamide as well as enzalutamide, in contrast to the mild CYP3A4 inducer darolutamide. Abiraterone is classified as a moderate inhibitor of the CYP2D6 isozyme, which catalyses the biotransformation of fewer drugs. As a consequence, abiraterone’s spectrum of interaction has to be interpreted differently. As pharmacotherapy approaches, it is helpful to understand the drug biotransformation pathways in more detail, in order to assess the extent of possible interactions that may necessitate the intensification of therapeutic monitoring, or dose modification or any therapeutic switch in time.

## Glossar

Abirateron: Abirateronacetat
Abi/P: Abirateronacetat und Prednison bzw. Prednisolon
ADT: Androgenentzugstherapie (androgen depreviation therapy)
ARI: Androgenrezeptor-Inhibitor
ARPI: Androgenrezeptor-Signalweg-Inhibitor
AUC: Fläche unter der Kurve (Area Under the Curve, beschreibt die Wirkstoffexposition über die Zeit)
BCRP: Breast Cancer Resistance Protein (auch bekannt als ABCG2=ATP-bindendes Kassetten-Superfamilien-G-Mitglied 2; ATP-binding cassette super-family G member 2)
CYP: Cytochrom-P450
DDI: Arzneimittelwechselwirkungen (drug-drug-interactions)
INN: internationaler Freiname
PgP: P-Glycoprotein (auch bekannt als ABCB1=ATP Binding Cassette Subfamily B Member 1)
SULT: Sulfotransferase
TCA: trizyklische Antidepressiva
UGT: UDP-Glucuronosyltransferase

## Einleitung

Die Anwendung von Androgenrezeptor-Signalweg-Inhibitoren (ARPI) der zweiten Generation, bestehend aus den Androgenrezeptor-Inhibitoren (ARI) Apalutamid, Darolutamid und Enzalutamid sowie dem CYP17-Inhibitor Abirateronacetat (in Kombination mit Prednison [Abi/P]), stellt sowohl beim hormonsensitiven metastasierten Prostatakarzinom (HSPC) als auch im Stadium der Kastrationsresistenz (CRPC) einen aktuell anerkannten, klinischen Standard dar ( Tab. 1 ). Die große Akzeptanz dieser Therapieoptionen ist mit ihrer nachweisbar hohen therapeutischen Effizienz verbunden 1 2 3 und durch die Möglichkeit der oralen Einnahme der Präparate begründet. Allerdings gehen von den verschiedenen ARI und Abi/P teilweise sehr unterschiedliche inhibitorische und induktive Effekte auf bestimmte Cytochrom-P450(CYP)-Isoenzyme und transmembranäre Effluxpumpen aus ( Tab. 2 ), die es im Rahmen einer Komedikation zu berücksichtigen gilt 4 5 6 7 . Insbesondere in der täglichen klinischen Pharmakotherapie von älteren und komorbiden Patienten ist häufig eine Polypharmazie mit Einnahme von 5–10 verschiedenen Arzneimitteln pro Tag zu beobachten 8 9 . Zu den am häufigsten eingesetzten Arzneimitteln zählen in diesem Zusammenhang u.a. orale Antidiabetika, Antihypertensiva, Antikoagulanzien, Thrombozytenaggregationshemmer, Lipidsenker, Analgetika, Protonenpumpeninhibitoren, miktionsbeeinflussende Arzneimittel und zahlreiche Psychopharmaka.

**Table:** **Tab. 1** Wichtige Eckdaten zum Einsatz der oralen Antiandrogene und Hormonantagonisten der 2. Generation 10 11 12 13 14 15 16 17 .

INN	Indikation*		Dosierung	Wirkmechanismus	Akkumulation‡	Penetration der Blut-Hirn-Schranke ^†^ 18 19	Zulassungsstudien
Abirateron ^#^ Verwendung in Kombination mit Prednison oder Prednisolon. 5 mg Prednisolon bzw. Prednison bei mHSCP. 10 mg Prednison bzw. Prednisolon bei mCRPC. 11	mHSPC	neu diagnostiziert, Hochrisiko ^∆^ , in Kombination mit ADT	einmal täglich 1000 mg oral (Nüchtern-Einnahme)	selektive Inhibition von 17α-Hydroxylase/C17,20-lyase (CYP17). Dies senkt in Kombination mit LHRH-Analoga oder Orchiektomie den Testosteronspiegel unter die Nachweisgrenze		ja	LATITUDE
	mCRPC	asymptomatischer oder mild symptomatischer Verlauf, nach Versagen der ADT, wenn eine Chemotherapie klinisch noch nicht indiziert ist					STAMPEDE COU-AA-302
		bei Fortschreiten der Erkrankung während oder nach einer Docetaxel-haltigen Chemotherapie					COU-AA-301
Apalutamid ^#,§^ 20	mHSPC	in Kombination mit ADT	einmal täglich 240 mg oral	Inhibition des ARs und dessen Signalweg: Hemmung der Bindung von Testosteron und anderen Androgenen an den AR Hemmung der Translokation des AR in den Nukleus Hemmung der AR-vermittelten Transkription	5,3-fach	ja	SPARTAN TITAN
	nmCRPC	bei nicht metastasiertem kastrationsresistentem Hochrisiko ^κ^ -PC					
Enzalutamid ^#^ 21	mHSPC	in Kombination mit ADT	einmal täglich 160 mg oral		8,3-fach	ja	ENZAMET
	mCRPC	asymptomatischer oder mild symptomatischer Verlauf, nach Versagen von ADT, wenn eine Chemotherapie klinisch noch nicht indiziert ist					
		bei Fortschreiten der Erkrankung während oder nach einer Chemotherapie mit Docetaxel					PREVAIL AFFIRM
	nmCRPC	bei nicht metastasiertem kastrationsresistentem Hochrisiko ^κ^ -PC					PROSPER
Darolutamid ^#^ 22 23	mHSPC	in Kombination mit Docetaxel und ADT	zweimal täglich 600 mg oral (=1200 mg pro Tag), in Kombination mit Docetaxel (75 mg/m ^2^ /kg)		2-fach	vernachlässigbar	ARASENS
		in Kombination mit ADT, derzeit noch nicht in Deutschland zugelassen					ARANOTE
	nmCRPC	bei nicht metastasiertem kastrationsresistentem Hochrisiko ^κ^ -PC	zweimal täglich 600 mg oral (=1200 mg pro Tag; Einnahme zu Mahlzeiten)				ARAMIS
* Abirateron, Apalutamid, Enzalutamid und Darolutamid sind zur Behandlung erwachsener Männer zugelassen ‡ Akkumulation: Anreicherung der Substanz im Körper ^†^ Ergebnisse aus präklinischen Untersuchungen ^#^ Während der Behandlung mit Abirateron, Enzalutamid und Darolutamid von Patienten, bei denen keine operative Kastration vorgenommen wurde, soll eine medikamentöse Kastration mit einem LHRH-Analogon durchgeführt werden. ^∆^ Hochrisiko (Abirateron): Vorliegen von mindestens 2 der folgenden 3 Risikofaktoren: Gleason-Score ≥8, Vorliegen von mindestens 3 Läsionen in der Knochenszintigrafie, Vorliegen von messbaren viszeralen Metastasen (ausgeschlossen Lymphknotenbefall) 11 . ^§^ Während der Behandlung mit Apalutamid von Patienten, bei denen keine operative Kastration vorgenommen wurde, soll eine medikamentöse Kastration mit einem GnRH-Analogon durchgeführt werden. ^κ^ Nicht metastasiertes kastrationsresistentes Hochrisiko-PC: Liegt vor, bei einem Befund nmCRPC, wenn die Dopplungszeit des prostataspezifischen Antigens ≤10 Monate beträgt 24 25 26 . ADT=Androgenentzugstherapie (androgen deprivation therapy); AR=Androgenrezeptor; CRPC=kastrationsresistentes Prostatakarzinom (castration resistant prostate cancer); CYP=Cytochrom-P450; GnRH=Gonadotropin-freisetzenden Hormonanalogon; HSPC=hormonsensitives Prostatakarzinom (hormone sensitive prostate cancer); INN=internationaler Freiname; LHRH=Luteinisierendes-Hormon-Releasing-Hormon; m=metastasierend (metastatic); nm=nicht metastasierend (non-metastatic); PC=Prostatakarzinom (prostate cancer)							

**Table:** **Tab. 2** Inhibitorische bzw. induktive Effekte ausgewählter Antiandrogene und Hormonantagonisten auf CYP-Isoenzyme und Effluxtransporter 4 5 6 7 .

INN	Inhibition/Induktion	Ausmaß der Wechselwirkung	Veränderung der Exposition von Modellsubstraten
Abirateron 11	CYP2D6-Inhibiton CYP2C8-Inhibition	moderat moderat	AUC (Dextromethorphan): +190% AUC (Pioglitazon): >+100%
Apalutamid 20	CYP3A-Induktion CYP2C19-Induktion CYP2C9-Induktion PgP-Induktion	stark stark moderat– leicht leicht	AUC (Midazolam): –92% AUC (Omeprazol): –85% AUC (Warfarin): –46% AUC (Fexofenadin): –30%
Darolutamid 22	CYP3A-Induktion BCRP-Inhibition	leicht stark	AUC (Midazolam): –29% AUC (Rosuvastatin): +400%
Enzalutamid 21	CYP3A-Induktion CYP2C19-Induktion CYP2C9-Induktion PgP-Inhibition	stark moderat moderat leicht	AUC (Midazolam): –86% AUC (Omeprazol): –70% AUC (S-Warfarin): –56% AUC (Digoxin): +33%
AUC=Fläche unter der Kurve (Area Under the Curve); BCRP=Breast Cancer Resistance Protein (syn.: ABCG2); CYP=Cytochrom-P450; INN=internationaler Freiname; PgP=P‑Glycoprotein (syn.: ABCB1) Anmerkungen zur Klassifizierung eines Induktors: Leichte Induktion (z.B. CYP3A): AUC-Abnahme der Komedikation (Midazolam) um ≥20 bis <50%; moderate Induktion: AUC-Abnahme um ≥50 bis <80%; potente (starke) Induktion: AUC-Abnahme um ≥80%. Leichte Inhibition (z.B. CYP2D6 oder CYP3A): AUC-Zunahme der Komedikation um das ≤2-fache; moderate Inhibition: AUC-Zunahme um das 2- bis 5-Fache; starke Inhibition: AUC-Zunahme um mehr als das 5-Fache.			

Bedauerlicherweise stehen nur für die wenigsten möglichen Arzneimittelkombinationen ( Tab. 3 **–14** ) klinisch-pharmakokinetische Studienergebnisse zur Verfügung. Daher müssen bis auf Weiteres Analogieschlüsse aus Untersuchungen mit Modellsubstraten in vivo gezogen werden, die aufgrund ihrer Biotransformationswege Ähnlichkeiten mit Substraten, die bisher geprüft wurden ( Tab. 2 ), zeigen 27 28 . Dabei ist der Umfang der Metabolisierung durch die entsprechenden CYP-Isoenzyme, die Bildung aktiver und/oder inaktiver Stoffwechselprodukte, aber auch die Belastbarkeit der publizierten Daten in der endgültigen Abschätzung einer klinisch relevanten Wechselwirkung oft eine gewisse Herausforderung 27 28 . So wird beispielsweise der α1-Blocker Tamsulosin in einer großen Übersicht nur als CYP2D6-Substrat geführt 8 , tatsächlich ist der Wirkstoff aber sowohl als CYP2D6- als auch CYP3A4-Substrat einzuordnen 9 . Somit erklären sich auch unterschiedliche Einschätzungen zwischen Bolek H et al. 8 und der vorliegenden Übersicht. In den im folgenden Text dargestellten Tabellen wurde deshalb mehrfach geprüft, welche CYP-Isoenzyme und Effluxpumpen in den Biotransformationswegen involviert sind, wohlwissend, dass es durch neue Erkenntnisse immer wieder zu einer Veränderung des Wissensstands kommen kann.

**Table:** **Tab. 3** Mögliche Auswirkungen verschiedener antiandrogen wirksamer Arzneimittel auf die Plasmakonzentrationen ausgewählter Antidiabetika (mod. nach 28 29 ).

Antidiabetika (Abbauwege)	Abirateron*	Apalutamid	Enzalutamid	Darolutamid
Acarbose (–)				
Dapagliflozin (–)				
Empagliflozin (–)				
Gliclazid (CYP2C9)		↓–↓↓	↓–↓↓	
Glimepirid (CYP2C9)		↓–↓↓	↓–↓↓	
Inkretinmimetika (z.B. Exenatid (–)				
Insuline (–)				
Metformin (–)				
Nateglinid (CYP2C9)		↓–↓↓	↓–↓↓	
Repaglinid (CYP2C8, 3A)	↑↑	↓–↓↓	↓–↓↓	
Pioglitazon (CYP2C8)	↑↑			
Saxagliptin (CYP3A)		↓↓↓	↓↓↓	↓
Sitagliptin (gering, PgP > CYP3A4, 2C8)		↓	↓	
CYP=Cytochrom-P450; PgP=P-Glycoprotein; WW=Wechselwirkungen; (–)=CYP- und PgP-unabhängige Clearance ↓: Folge einer schwachen Induktion; ↓↓: Folge einer mäßigen Induktion; ↓↓↓: Folge einer starken Induktion * Abirateron stellt die eigentliche Wirkform des verabreichten Abirateronacetats dar. Der CYP17-Inhibitor wird durchgängig mit dem Glucocorticoid Prednison (5–10 mg/Tag) kombiniert, das in dieser Dosis allerdings zu keinen klinisch-pharmakokinetischen Wechselwirkungen führt				

Aus den sich hieraus ergebenden möglichen Arzneimittelinteraktionen ( Tab. 3 **–14** ) kann für den individuellen Patienten zum einen durch zu hohe Wirkstoffspiegel der Komedikation (↑↑↑) ein erhöhtes Risko für unerwünschte Nebenwirkungen, zum anderen durch zu niedrige Wirkstoffspiegel der Komedikation (↓↓↓) eine reduzierte Effektivität der Komedikation abgeleitet werden 10 30 31 . Da neben der Verzögerung der Krankheitsprogression vor allem die Verträglichkeit den zentralen Faktor für die Lebensqualität unter einer antitumoralen Therapie darstellt 32 , ist eine gezielte, kritische Analyse des Komedikations- und Interaktionsprofils eines Patienten vor Beginn dieser Therapie sinnvoll 33 34 .

Die Beurteilung der Relevanz möglicher Interaktionen von Komedikation und ARI bzw. Abi/P ist eine komplexe, interdisziplinäre Aufgabe, die eine große Herausforderung in der klinischen Routine darstellt 14 33 35 . Daher soll der folgende Artikel Orientierung und Hilfestellung bieten, um rechtzeitig potenziell kritische Komedikationen zu identifizieren und ggf. Therapiealternativen zu diskutieren. Die folgenden grafischen Darstellungen ( Tab. 3 **–14** ) versuchen über die aufgeführten Pfeile die Wahrscheinlichkeit des Ausmaßes einer klinisch-pharmakokinetischen Erhöhung oder Erniedrigung von Plasmaspiegeln der Komedikation etwas zu präzisieren.

## Antidiabetika

Bei den oralen Antidiabetika werden vor allem die Sulfonylharnstoffe (z.B. Glimepirid) und die Glinide über verschiedene CYP-Isoenzyme verstoffwechselt und inaktiviert 36 . Bei Saxaglipitin ist es ausschließlich das Cytochrom-P450–3A4-Isoenzym. Abhängig von der Stärke der CYP2C- oder CYP3A4-Enzyminduktion ist deshalb mit einer Abnahme der blutzuckersenkenden Wirkung bei z.B. Saxagliptin zu rechnen, während unter Abi/P (moderater CYP2C8-Inhibitor) eine Wirkungsverstärkung bei Gliniden auftreten kann. Daher empfiehlt sich eine engmaschigere Überprüfung des Blutzuckerspiegels und gegebenenfalls eine Umstellung auf Wirkstoffe, die nicht über das CYP-System verstoffwechselt werden ( Tab. 3 ).

## Orale Antikoagulanzien

Der Vitamin-K-Antagonist Phenprocoumon wird vor allem über CYP3A4, weniger über CYP2C9 verstoffwechselt 37 , während das strukturverwandte RS-Warfarin, das vor allem in angloamerikanischen Ländern zum Einsatz kommt, über CYP3A, 2C9 und 1A2 biotransformiert wird 38 . Folglich ist unter Apalutamid bzw. Enzalutamid eine deutliche Senkung der International Normalized Ratio (INR)-Werte von Phenprocoumon bzw. Warfarin zu erwarten ( Tab. 4 ).

**Table:** **Tab. 4** Mögliche Auswirkungen verschiedener antiandrogen wirksamer Arzneimittel auf die Plasmakonzentrationen ausgewählter Antikoagulanzien und Thrombozytenaggregationshemmer (mod. nach 24 28 39 40 ).

Antikoagulanzien (Abbauwege)	Abirateron	Apalutamid	Enzalutamid	Darolutamid
Apixaban (CYP3A, BCRP > PgP)		↓↓↓	↓↓↓	↓
Clopidogrel† (Prodrug) (CYP2C19 u.a.)		↓↓↓† (Prodrug-Aktivierung ↑↑)	↓↓↓† (Prodrug-Aktivierung ↑↑)	
Dabigatran (PgP)		↓–↓↓ (PgP)	↑ (PgP)	
Edoxaban (PgP)		↓–↓↓ (PgP)	↑ (PgP)	
Phenprocoumon (CYP3A > 2C9)		↓↓↓	↓↓↓	↓
Prasugrel* (Prodrug) CYP3A, 2B6 u.a.				
Rivaroxaban (CYP3A, BCRP, PgP)		↓↓↓	↓↓↓	↓
Ticagrelor (CYP3A)		↓↓↓	↓↓↓	↓
Tinzaparin (NHM) (–)				
RS-Warfarin (CYP3A, 2C9,1A2)		↓↓↓	↓↓↓	↓
BCRP=Breast Cancer Resistance Protein; CYP=Cytochrom-P450; NHM=niedermolekulare Heparine; PgP=P-Glycoprotein; WW=Wechselwirkungen; (–)=CYP- und PgP-unabhängige Clearance ↓: Folge einer schwachen Induktion; ↓↓: Folge einer mäßigen Induktion; ↓↓↓: Folge einer starken Induktion * keine relevante Veränderung der pharmakodynamischen Eigenschaften unter dem Einfluss von CYP3A-Induktoren zu erwarten † Die Abnahme an Clopidogrel ist mit einer stärkeren Bioaktivierung und Bildung aktiver Metaboliten verbunden, was mit einer stärkeren Thrombozytenaggregationshemmung (TAH) einhergehen kann, wenn man Analogschlüsse zum CYP3A/2C-Induktor Rifampicin zieht 41 .				

Bei den direkten oralen Antikoagulanzien (DOAK) werden Apixaban und Rivaroxaban durch CYP3A metabolisiert und inaktiviert 12 13 42 . Als Effluxpumpe spielt beim Apixaban wahrscheinlich vor allem BCRP (ABCG2), beim Rivaroxaban BCRP und PgP (ABCB1) eine Rolle 43 44 . Apalutamid und Enzalutamid sollten nicht mit den CYP3A-Substraten Apixaban bzw. Rivaroxaban kombiniert werden ( Tab. 3 ). Darolutamid induziert CYP3A nur leicht und hemmt BCRP. Daher dürfte es keine klinisch relevanten pharmakokinetischen Veränderungen bei Apixaban und Rivaroxaban verursachen ( Tab. 4 ).

Edoxaban und das Prodrug Dabigatranetexilat sind keine relevanten CYP-Substrate. Stattdessen ist die Effluxpumpe PgP besonders für die Elimination von Dabigatranetexilat und, in etwas geringerem Ausmaß, von Edoxaban entscheidend 45 46 . Da Apalutamid im Rahmen länger andauernder Therapien auch PgP induzieren kann ( Tab. 2 ), ist eine Abnahme der entsprechenden Plasmaspiegel und der antikoagulatorischen Wirkung nicht auszuschließen. Beim Edoxaban bietet sich im Zweifel ein anti-Xa-Monitoring der Talspiegel vor der nächsten Einnahme an, um das Ausmaß einer reduzierten antikoagulatorischen Wirkung besser abschätzen zu können.

Auch beim Enzalutamid wurden anfangs – basierend auf In.vitro-Daten – deutliche PgP-induzierende Eigenschaften angenommen. Allerdings haben die kürzlich veröffentlichten Studienergebnisse von Poondru et al. diese Einschätzung nicht bestätigt ( Tab. 2 ) 47 .

## Thrombozytenaggregationshemmer

Der Thrombozytenaggregationshemmer (TAH) Acetylsalicylsäure wird über das Enzym UGT1A6 metabolisiert 48 . Da UGT1A6 weder durch die Antiandrogene noch durch Abi/P beeinflusst wird, kann Acetylsalicylsäure unter pharmakokinetischen Gesichtspunkten vergleichsweise problemlos mit diesen Arzneimitteln kombiniert werden.

Clopidogrel ist der am häufigsten in der klinischen Praxis eingesetzte ADP-Antagonist und ein Prodrug, das ganz wesentlich durch CYP2C19 bioaktiviert wird 49 . Einer der dabei entstehenden Metabolite, Clopidogrel-acyl-β-D-glucuronid, weist moderate CYP2C8-inhibierende Eigenschaften auf. Daher empfiehlt die Fachinformation des CYP2C8-Substrats Enzalutamid im Rahmen einer begleitenden Therapie mit Clopidogrel eine Halbierung der Enzalutamid-Dosis 21 ! Klinisch-pharmakokinetische Studienergebnisse zu dieser Kombination fehlen derzeit noch. Eine Halbierung der Enzalutamid-Dosis, wie vorgegeben, soll zu Enzalutamid-Konzentrationen im Plasma führen, wie sie ohne Clopidogrel zu erwarten wären. Gleichzeitig ist mit einer stärkeren Bioaktivierung von Clopidogrel zu rechnen, sodass eine ausgeprägtere TAH die Folge sein kann. Will man dieser relativ komplexen pharmakokinetischen Wechselwirkung aus dem Weg gehen, sind aus klinisch-pharmakokinetischen Überlegungen heraus Abi/P bzw. Darolutamid zu bevorzugen ( Tab. 2 ).

Der ADP-Antagonist Prasugrel ist ebenfalls ein Prodrug, wird aber nicht über CYP2C19, sondern über die Isoenzyme CYP2B6 und CYP3A aktiviert 38 50 . Studien mit dem CYP3A-Induktor Rifampicin konnten unter der Kombination keine klinisch relevante Steigerung der TAH erkennen, sodass auch unter Apalutamid und Enzalutamid das Blutungsrisiko nicht verändert sein dürfte 50 . Allerdings sind bei Prasugrel die Indikationseinschränkungen und Kontraindikationen, wie beispielsweise ein Schlaganfall in der Anamnese 50 , zu beachten. Da Ticagrelor im Gegensatz zu Clopidogrel und Prasugrel kein Prodrug ist, führt eine CYP3A-Induktion zu einer direkten Abnahme der TAH-Wirkung ( Tab. 4 ) 51 . Dieser Wechselwirkung geht man mit Abi/P bzw. Darolutamid aus dem Weg.

Keine pharmakokinetischen und -dynamischen Wechselwirkungen sind wiederum zwischen den Antiandrogenen und den niedermolekularen Heparinen (NMH) zu erwarten, da letztere CYP-unabhängig verstoffwechselt und ausgeschieden werden 52 .

## Lipidsenker

Bei den 3-Hydroxy-3-Methylglutaryl-Coenzym-A(HMG-CoA)-Reduktasehemmern („Statinen“) Atorvastatin und Simvastatin handelt es sich um Substrate von CYP3A, Fluvastatin ist ein CYP2C9-Substrat. Daher ist unter Apalutamid und Enzalutamid teilweise mit einer erheblichen Abnahme der Plasmaspiegel dieser Statine zu rechnen, sodass im Rahmen einer entsprechenden Kombination die LDL-Senkungen unter Standarddosen weit hinter den Erwartungen zurückbleiben können ( Tab. 5 ).

**Table:** **Tab. 5** Mögliche Auswirkungen verschiedener antiandrogen wirksamer Arzneimittel auf die Plasmakonzentrationen ausgewählter Lipidsenker (mod. nach 27 28 53 54 55 ).

Lipidsenker (Abbauwege)	Abirateron	Apalutamid	Enzalutamid	Darolutamid
Atorvastatin (CYP3A, PgP)		↓↓↓	↓↓↓	↓
Bempedoinsäure (UGT)				
Ezetimib (UGT)				
Fluvastatin (CYP2C9 > 3A, 2C8, BCRP)		↓↓↓	↓↓↓	
Icosapent (–) bzw. Colesevelam (–)				
Pravastatin (SULT)				
Rosuvastatin (BCRP)				↑↑↑
Simvastatin (CYP3A, PgP, BCRP)		↓↓↓	↓↓↓	↓–↓↓
BCRP=Breast Cancer Resistance Protein; CYP=Cytochrom-P450; PgP=P-Glycoprotein ; SULT (Sulfotransferasen), UGT=UDP-Glucuronosyltransferase; WW=Wechselwirkungen; (–)=CYP- und PgP-unabhängige Clearance ↑↑↑: Folge einer starken Inhibition, ↓: Folge einer schwachen Induktion; ↓–↓↓: Folge einer schwachen bis mäßigen Induktion; ↓↓↓: Folge einer starken Induktion				

Pravastatin ist ein Substrat der Sulfotransferasen 33 und daher weniger interaktionsanfällig. Allerdings ist der Wirkstoff mit einer Dosis von maximal 40 mg pro Tag nur für moderate LDL-Senkungen von ca. 36% gegenüber dem Ausgangswert geeignet 14 .

Rosuvastatin wird kaum über CYP2C9 verstoffwechselt, sondern vorwiegend über die Effluxpumpe BCRP (syn.: ABCG2) ausgeschieden 56 . Da Darolutamid ein spezifischer BCRP-Inhibitor ist, kommt es in Verbindung mit Rosuvastatin zu einem Anstieg des Statins im Plasma um das 5-Fache. Bei einer entsprechenden Kombination ist deshalb die Rosuvastatin-Dosis auf 5 mg pro Tag zu begrenzen und die LDL-Senkung bei Bedarf engmaschiger zu überwachen 26 .

Die Lipidsenker Ezetimib, Bempedoinsäure und Colesevelam sind keine CYP-Substrate 35 41 55 , Veränderungen der Plasmaspiegel unter Apalutamid und Enzalutamid sind deshalb unwahrscheinlich. Dasselbe gilt für Icosapent, einem Derivat der Omega-3-Fettsäure Eicosapentaensäure (EPA) 27 .

## Analgetika

Unter den nicht-steroidalen Analgetika/Antirheumatika/Antiphlogistika (NSAR) befinden sich sowohl einige CYP2C9-Substrate (z.B. Ibuprofen, Celecoxib, Diclofenac) als auch CYP3A-Substrate wie Etoricoxib 28 57 . Das Spektrum an zu erwartenden Wechselwirkungen mit den Antiandrogenen variiert deshalb von Substanz zu Substanz ( Tab. 6 ).

**Table:** **Tab. 6** Mögliche Auswirkungen verschiedener antiandrogen wirksamer Arzneimittel auf die Plasmakonzentrationen ausgewählter Analgetika (mod. nach 2 8 28 42 57 58 59 ).

Analgetikum (Abbauwege)	Abirateron	Apalutamid	Enzalutamid	Darolutamid
Buprenorphin (CYP3A)		↓↓↓	↓↓↓	↓
Celecoxib (CYP2C9)		↓↓	↓↓	
Codein (Prodrug) (UGT2B7, CYP2D6 >3A)	↑			
Diclofenac (CYP2C9 u.a.)*		↓–↓↓	↓–↓↓	
Dihydrocodein (CYP3A > 2D6)	↑	↓↓	↓↓	↓
Etoricoxib (CYP3A)		↓↓↓	↓↓↓	↓
Fentanyl (CYP3A)		↓↓↓	↓↓↓	↓
Hydromorphon (UGT)				
Ibuprofen (CYP2C9, 2C8)		↓↓	↓↓	
Levomethadon (CYP3A > 2B6, 2D6, 2C19)		↓↓↓	↓↓↓	↓
Metamizol (CYP2C9, 2C19, NAT u.a.)		↓–↓↓	↓–↓↓	
Morphin (UGT2B7)				
Naproxen (UGT2B7)				
Oxycodon/Naloxon (CYP3A > 2D6)		↓↓↓	↓↓↓	↓
Paracetamol (CYP2E1, UGT1A1)				
Tapentadol (UGT > CYP2C9,2C19)		↓	↓	
Tilidin/Naloxon (Prodrug) (CYP3A, 2C19)		↓↓	↓↓	↓
Tramadol (Prodrug) (CYP2D6 > 3A, 2B6)	↑			
CYP=Cytochrom-P450; NAT (N-Acetyltransferase), UGT=UDP-Glucuronosyltransferase; WW=Wechselwirkungen; (–)=CYP- und PgP-unabhängige Clearance ↑: Folge einer schwachen Inhibition, ↓: Folge einer schwachen Induktion; ↓–↓↓: Folge einer schwachen bis mäßigen Induktion; ↓↓: Folge einer mäßigen Induktion; ↓↓↓: Folge einer starken Induktion, * Metabolismus in partiellem bzw. geringem Umfang				

Schwer vorauszusehen sind die Auswirkungen eines CYP2C19/2C9-Induktors auf das klinisch-pharmakokinetische und analgetische Profil von Metamizol (syn.: Novaminsulfon), eines der am häufigsten eingesetzten Schmerzmittel in Deutschland. Das Prodrug wird nach oraler Einnahme schnell nicht-enzymatisch in MAA (4-Methylaminoantipyrin) hydrolysiert. Über CYP2C9, CYP2C19 und CYP1A2, aber auch die N-Acetyltransferase erfolgt die Bildung der ebenfalls aktiven Metabolite FAA (4-Formylantipyrin) und AA (4-Aminoantipyrin). Alle drei, MAA, FAA und AA, können mit der Arachidonsäure Amide bilden und so ihre analgetische Wirkung entfalten. Da das hierfür verantwortliche Enzym, die Fettsäureamidhydrolase (FAAH) vor allem im ZNS und in der Leber exprimiert wird, besitzt Metamizol nur eine mäßige antiphlogistische Wirkung (z.B. Bei Gelenkschmerzen), wirkt aber gut analgetisch und antipyretisch im ZNS sowie spasmolytisch auf die glatte Muskulatur (z.B. Gallengänge).

Bei Probanden mit CYP2C19*17/*17-Allelen (Ultrarapid-Metabolizer mit hoher konstitutiver CYP2C19-Aktivität) waren geringere Mengen an aktiven Metaboliten zu erkennen, da sie wahrscheinlich weiter verstoffwechselt wurden. Ein ähnliches Bild ist auch unter Apalutamid oder Enzalutamid vorstellbar. Zu beachten sind beim Metamizol allerdings die grundsätzlichen Nebenwirkungen wie die potenzielle Hepatotoxizität, Agranulozytosen und Überempfindlichkeitsreaktion, sodass generell die Therapiedauer begrenzt werden sollte 8 28 59 .

Bei den Opiaten und Opioiden (synthetischen Ursprungs) sind es vor allem Fentanyl, Buprenorphin, Methadon, Oxycodon sowie Tilidin, die über CYP3A verstoffwechselt werden 43 53 60 , sodass ihre Plasmaspiegel durch Apalutamid bzw. Enzalutamid deutlich abfallen können ( Tab. 6 ).

Hydromorphon ist unter den Opiaten/Opioiden in diesem Zusammenhang am wenigsten interaktionsanfällig 34 – ähnlich wie Morphin. Allerdings ist letzteres bei eingeschränkter Nierenfunktion aufgrund seiner pharmakodynamisch aktiven Glucuronide schlechter steuerbar.

Für die Bioaktivierung der Prodrugs Codein und Tramadol spielt das Isoenzym CYP2D6 eine wichtige Rolle 60 61 . Da Abi/P ein moderater Hemmstoff dieses Isoenzyms ist, ist eine Beeinträchtigung der analgetischen Wirkung im Rahmen einer Kombinationstherapie möglich, während die Plasmaspiegel an unverändertem Codein und Tramadol ansteigen ( Tab. 6 ).

Bei Tilidin handelt es sich ebenfalls um ein Prodrug, das über CYP3A und CYP2C19 bioaktiviert wird 53 . Da auch das inaktive Folgeprodukt Bisnortilidin über diese Isoenzyme entsteht, ist in der Gesamtbetrachtung wahrscheinlich von einer Wirkungsabschwächung unter Apalutamid und Enzalutamid auszugehen.

## Opiatantagonisten

Von den verfügbaren Opiatantagonisten werden Methylnaltrexon s.c. und Naloxon CYP-unabhängig verstoffwechselt, sodass keine klinisch relevanten Interaktionen mit den ARI und Abi/P zu erwarten sind. Hingegen werden Naloxegol und Naldemedine via CYP3A4 zu inaktiven Metaboliten verstoffwechselt, sodass deren antagonistischen Effekte auf eine opioidassoziierte Obstipation deutlich abgeschwächt sein dürften, wenn Apalutamid oder Enzalutamid gleichzeitig zum Einsatz kommen 52 .

## Immunsuppressiva

Die Calcineurin- bzw. m-TOR-Inhibitoren Cyclosporin, Tacrolimus, Everolimus und Sirolimus werden nach oraler Einnahme in erster Linie über CYP3A verstoffwechselt und inaktiviert 28 61 62 . Daher kann es in Verbindung mit CYP3A-Induktoren wie Apalutamid oder Enzalutamid zu einer außerordentlich starken Abnahme des Plasmaspiegels dieser Immunsuppressiva kommen ( Tab. 7 ). Mit Darolutamid ist eine – wenn auch deutlich geringere – Reduktion der Exposition dieser Wirkstoffe zu erwarten, sodass ein engmaschigeres therapeutisches Drug Monitoring (TDM) empfohlen wird.

**Table:** **Tab. 7** Mögliche Auswirkungen verschiedener antiandrogen wirksamer Arzneimittel auf die Plasmakonzentrationen ausgewählter Immunsuppressiva (mod. nach 28 46 61 62 ).

Immunsuppressiva (Abbauwege)	Abirateron	Apalutamid	Enzalutamid	Darolutamid
Kortikosteroide (z.B. Dexamethason) (CYP3A)		↓↓↓	↓↓↓	↓
Cyclosporin (CYP3A, PgP)		↓↓↓	↓↓↓	↓
Everolimus (CYP3A, 2C8 PgP)		↓↓↓	↓↓↓	↓
MMF/MPA (UGT1A9)				
Sirolimus (CYP3A, PgP)		↓↓↓	↓↓↓	↓
Tacrolimus (CYP3A, PgP)		↓↓↓	↓↓↓	↓
CYP=Cytochrom-P450; MMF=Mycophenolat mofetil; MPA=Mycophenolsäure; PgP=P-Glycoprotein; UGT=UDP-Glucuronosyltransferase; WW=Wechselwirkungen ↓: Folge einer schwachen Induktion; ↓↓↓: Folge einer starken Induktion				

Mycophenolatmofetil (MMF) bzw. Mycophenolsäure (MPA) werden über die Isoenzyme UGT2B7 und UGT1A9 glucuronidiert und verstoffwechselt 28 58 , eine Interaktion mit den Antiandrogenen ist deshalb unwahrscheinlich.

Glucocorticoide wie Dexamethason, Prednisolon aber auch Budesonid zählen zu den CYP3A-Substraten 4 57 59 , sodass eine deutliche Abnahme ihrer Plasmaspiegel und immunsuppressiven Wirkung unter Apalutamid und Enzalutamid zu erwarten ist ( Tab. 7 ).

## Miktionsbeeinflussende Arzneimittel

Viele der anticholinerg (antimuscarinerg) wirksamen Pharmaka unter den miktionsbeeinflussenden Arzneimitteln sind CYP3A-Substrate ( Tab. 8 ). Der Umfang des First-Pass-Effekts (d.h. metabolische Passage durch Darm und Leber) kann, abhängig von der jeweiligen Substanz, erheblich sein, wie beispielsweise beim Oxybutinin, das nach oraler Gabe mit einer absoluten Bioverfügbarkeit von nur ca. 6% verbunden ist. Flavoxat und Trospiumchlorid bieten in diesem Zusammenhang gewisse Vorteile, weil sie praktisch nicht über das CYP-System verstoffwechselt werden 63 64 .

**Table:** **Tab. 8** Mögliche Auswirkungen verschiedener antiandrogen wirksamer Arzneimittel auf die Plasmakonzentrationen ausgewählter miktionsbeeinflussender Arzneimittel, inkl. einiger α-Blocker (mod. nach 18 28 49 60 65 66 ).

miktionsbeeinflussende Arzneimittel (Abbauwege)	Abirateron	Apalutamid	Enzalutamid	Darolutamid
Alfuzosin (CYP3A4)		↓↓↓	↓↓↓	↓
Darifenacin (PgP, CYP2D6, 3A)	↑	↓↓↓	↓↓↓	↓
Fesoterodin (CYP3A, 2D6)	↑	↓↓↓	↓↓↓	↓
Flavoxat (Esterasen)				
Mirabegron (AmidH, UGT, CYP3A, 2D6)		↓	↓	
Oxybutinin (CYP3A)		↓↓↓	↓↓↓	
Propiverin (CYP3A)		↓↓↓	↓↓↓	↓
Solifenacin (CYP3A)		↓↓↓	↓↓↓	↓
Tamsulosin (CYP3A4, 2D6)	↑	↓↓↓	↓↓↓	↓
Terazosin (CYP3A)		↓↓↓	↓↓↓	↓
Tolterodin (CYP2D6 > 3A)	↑	↓–↓↓	↓–↓↓	
Trospiumchlorid (–)				
AmidH=Amidhydrolasen, CYP=Cytochrom-P450; PgP=P-Glycoprotein; WW=Wechselwirkungen ↑: Folge einer schwachen Inhibition; ↓: Folge einer schwachen Induktion; ↓↓: Folge einer mäßigen Induktion; ↓↓↓: Folge einer starken Induktion				

Da Darifenacin, Fesoterodin und Tolterodin über CYP2D6 verstoffwechselt werden ( Tab. 8 ), ist ein Anstieg der jeweiligen Plasmaspiegel unter Abirateron als Komedikation zu erwarten.

Bei den α-Blockern Alfuzosin, Tamsulosin und Terazosin spielt das CYP3A4 eine dominierende Rolle im Metabolismus 5 15 54 , sodass deren Plasmaspiegel unter Apalutamid bzw. Enzalutamid deutlich vermindert sein können.

## Magensäurereduzierende Arzneimittel

Bei den Protonenpumpeninhibitoren (PPI) werden vor allem Omeprazol und Esomeprazol, gefolgt von Pantoprazol über CYP2C19 verstoffwechselt und inaktiviert. Für die Biotransformation von Lansoprazol und Rabeprazol nimmt die Bedeutung dieses Isoenzyms sukzessive ab, sodass unter einer Komedikation mit CYP2C19-Induktoren unterschiedlich starke Auswirkungen auf die Plasmaspiegel dieser Wirkstoffe zu erwarten sind ( Tab. 9 ) 62 . Etwa 65–70% einer oral verabreichten Dosis des H2-Antihistaminikums Famotidin werden in unveränderter Form über den Urin ausgeschieden, der Rest wird als Sulfoxid eliminiert 65 . Deshalb sind relevante Wechselwirkungen mit CYP3A/2C9/2C19-Induktoren eher unwahrscheinlich.

**Table:** **Tab. 9** Mögliche Auswirkungen verschiedener antiandrogen wirksamer Arzneimittel auf die Plasmakonzentrationen ausgewählter magensäurereduzierender Arzneimittel (mod. nach 28 67 ).

Protonenpumpenhemmer (Abbauwege)	Abirateron	Apalutamid	Enzalutamid	Darolutamid
Dexlansoprazol (CYP2C19, kaum CYP3A4)		↓–↓↓	↓–↓↓	
Esomeprazol (CYP2C19)		↓↓–↓↓↓	↓↓–↓↓↓	
Famotidin (–)				
Lansoprazol (CYP2C19, kaum CYP3A4)		↓–↓↓	↓–↓↓	
Omeprazol (CYP2C19)		↓↓–↓↓↓	↓↓–↓↓↓	
Pantoprazol (CYP2C19, partiell CYP3A4)		↓↓	↓↓	
Rabeprazol (CYP2C19, kaum CYP3A4)		↓–↓↓	↓–↓↓	
CYP=Cytochrom-P450; WW=Wechselwirkungen; (–)=CYP- und PgP-unabhängige Clearance ↓: Folge einer schwachen Induktion; ↓–↓↓: Folge einer schwachen bis mäßigen Induktion; ↓↓: Folge einer mäßigen Induktion; ↓↓–↓↓↓: Folge einer mäßigen bis starken Induktion; ↓↓↓: Folge einer starken Induktion				

## Antihypertensiva und Hemmstoffe des Renin-Angiotensin-Aldosteron-Systems (RAAS)

Unter den Antihypertensiva sind es vor allem die Kalziumantagonisten vom Dihydropyridin-Typ (z.B. Amlodipin, Lercanidipin, Felodipin), die sehr umfangreich über CYP3A metabolisiert und inaktiviert werden ( Tab. 10 ) 6 16 68 . Im Rahmen einer Komedikation mit Apalutamid oder Enzalutamid ist deshalb mit unzureichenden Plasmaspiegeln unter Normdosen zu rechnen. Inwieweit in solchen Fällen durch eine Dosissteigerung des Antihypertensivums bei gleichzeitig engmaschigerer Blutdruckkontrolle eine Kombination dennoch möglich ist, wurde bisher nicht weitergehend untersucht.

**Table:** **Tab. 10** Mögliche Auswirkungen verschiedener antiandrogen wirksamer Arzneimittel auf die Plasmakonzentrationen ausgewählter Antihypertensiva (mod. nach 28 45 55 56 69 ).

Antihypertensiva (Abbauweg)	Abirateron	Apalutamid	Enzalutamid	Darolutamid
Amlodipin (CYP3A)		↓↓↓	↓↓↓	↓
Bisoprolol (CYP3A > 2D6)	↑	↓↓	↓↓	(↓)
Candesartan (–)				
Carvedilol (CYP2D6, 2C9)	↑↑	↓	↓	
Doxazosin (CYP3A4 > 2D6, 2C9)		↓↓	↓↓	↓
Enalapril (–)				
Felodipin (CYP3A)		↓↓↓	↓↓↓	↓–↓↓
Furosemid (–)				
Lercanidipin (CYP3A)		↓↓↓	↓↓↓	↓–↓↓
Metoprolol (CYP2D6)	↑↑			
Nebivolol (CYP2D6)	↑↑*			
Olmesartan (–)				
Ramipril (–)				
Sacubitril/Valsartan (–)				
Eplerenon (CYP3A)		↓–↓↓	↓–↓↓	
Losartan (CYP3A)		↓	↓	
Spironolacton (–)				
Torasemid (CYP2C9)		↓↓	↓↓	
CYP=Cytochrom-P450; WW=Wechselwirkungen; (–)=CYP- und PgP-unabhängige Clearance *Da die antihypertensive Wirkung aus der Summe aus Ausgangsstoff und Metaboliten resultiert, ist der Anstieg unter Abi/P klinisch nicht relevant. ↑: Folge einer schwachen Inhibition ; ↑↑: Folge einer mäßigen Inhibition; ↓: Folge einer schwachen Induktion; ↓–↓↓: Folge einer schwachen bis mäßigen Induktion; ↓↓: Folge einer mäßigen Induktion; ↓↓↓: Folge einer starken Induktion				

ACE-Hemmer (z.B. Ramipril, Enalapril) sind keine CYP3A-Substrate 17 66 . Auch der AT-II-Antagonist Candesartan wird CYP-unabhängig eliminiert 70 . Losartan und sein ebenfalls aktiver Metabolit werden partiell über CYP3A4 abgebaut, der Einfluss von CYP3A-Induktoren ist deshalb kaum klinisch relevant. Bei einigen β‑Blockern spielt das CYP-Isoenzym CYP2D6 eine relevante Rolle für die Biotransformation, allerdings nicht bei allen Vertretern ( Tab. 10 ). Daher sollten bei Metoprolol, Carvedilol oder Propranolol unter dem moderaten CYP2D6-Inhibitor Abirateron engmaschigere Blutdruckkontrollen erfolgen und im Zweifel die Dosis reduziert werden. Bisoprolol, das vor allem über CYP3A4 metabolisiert wird 55 , dürfte wiederum interaktionsanfälliger gegenüber Apalutamid und Enzalutamid sein, sodass niedrigere Plasmaspiegel an Bisoprolol zu erwarten sind. Nebivolol, dessen Metabolit (via CPY2D6 gebildet) ebenfalls antihypertensiv aktiv ist 28 , kann ohne Dosismodifikation mit Abi/P kombiniert werden.

Bei den Schleifendiuretika wird Furosemid primär unverändert renal eliminiert 71 . Torasemid wird dagegen vor allem über CYP2C9 verstoffwechselt 72 , sodass hier eine Abnahme der Plasmaspiegel und möglicherweise auch der diuretischen Wirkung unter Apalutamid oder Enzalutamid eintreten kann. Der α-Blocker Doxazosin ist ein CYP3A-Substrat 44 , sodass mit einem potenten CYP3A-Induktor eine Reduktion der Plasmakonzentration zu erwarten ist ( Tab. 10 ).

## Antipsychotika

Bei den Antipsychotika (früher: Neuroleptika) spielen substanzspezifisch verschiedene CYP‑Isoenzyme in der Biotransformation eine wichtige Rolle ( Tab. 11 ). Ein moderater CYP2D6-Inhibitor wie Abirateron kann daher substanzabhängig einen mehr oder weniger starken Anstieg der Plasmaspiegel verursachen (z.B. Thioridazin > Haloperidol u.a.), vor allem wenn nicht noch weitere CYP-Isoenzyme an der Biotransformation beteiligt sind. Bei einigen Vertretern der Antipsychotika kann die QT-Zeit verlängert sein. Dies muss im Rahmen eines Anstiegs der Plasmakonzentrationen unbedingt beachtet werden (z.B. bei Thioridazin 29 ).

**Table:** **Tab. 11** Mögliche Auswirkungen verschiedener antiandrogen wirksamer Arzneimittel auf die Plasmakonzentrationen ausgewählter Antipsychotika (mod. nach 21 28 70 ).

Antipsychotika (Abbauwege)	Abirateron	Apalutamid	Enzalutamid	Darolutamid
Amisulprid (–)				
Aripiprazol (CYP3A, 2D6)	↑	↓↓↓	↓↓↓	↓
Asenapin (UGT1A4, CYP1A2)				
Clozapin (CYP1A2, 2C19 > 3A)		↓–↓↓	↓–↓↓	
Haloperidol (CYP2D6 > 3A u.a.)	↑ (QT)	↓–↓↓	↓–↓↓	↓
Levomepromazin (CYP2D6)	↑↑ (QT)			
Olanzapin (CYP1A2)				
Paliperidon (–)				
Quetiapin (CYP3A)		↓↓↓	↓↓↓	
Risperidon (CYP2D6 > 3A)	↑↑	↓↓	↓↓	
Thioridazin (CYP2D6)	↑↑ (QT)			
CYP=Cytochrom-P450; PgP=P-Glycoprotein; QT=QT-Zeit; UGT=UDP-Glucuronosyltransferase; WW=Wechselwirkungen; (–)=CYP- und PgP-unabhängige Clearance ↑: Folge einer schwachen Inhibition ; ↑↑: Folge einer mäßigen Inhibition; ↓: Folge einer schwachen Induktion; ↓–↓↓: Folge einer schwachen bis mäßigen Induktion; ↓↓: Folge einer mäßigen Induktion; ↓↓↓: Folge einer starken Induktion				

Bei CYP3A-Substraten wie Aripiprazol, Quetiapin oder Risperidon können in Verbindung mit Apalutamid oder Enzalutamid wiederum subtherapeutische Konzentrationen die Folge sein ( Tab. 11 ). Empirische Dosissteigerungen sind in solchen Fällen nur unter engmaschiger Überwachung möglich, wenn überhaupt. In psychiatrischen Zentren sind allerdings für viele Psychopharmaka inzwischen Blutspiegelbestimmungen möglich (TDM), die eine zielgerichtete Dosistitration erlauben 51 70 .

Der Wirkstoff Melperon, der sehr häufig in der klinischen Praxis im Einsatz ist, ist bis heute in seinen Biotransformationswegen nicht genau charakterisiert worden, sodass aktuell keine Interaktionsrisiken mit den ARI und Abi/P formuliert werden können.

## Antidepressiva vom SSRI- und NaSRI-Typ

Selektive Serotoninwiederaufnahmehemmer (SSRI) bzw. Serotonin/Noradrenalin-Wiederaufnahmehemmer (NaSRI) spielen in der Behandlung von Depressionen weltweit eine wichtige Rolle. Für (Es)Citalopram oder Sertalin, sind deutliche Wirkstoffabnahmen im Plasma unter Apalutamid bzw. Enzalutamid zu erwarten. Für CYP2D6- bzw. CYP1A2-Substrate wie Duloxetin, Fluoxetin und Paroxetin sind mit diesen Antiandrogenen dagegen keine klinisch relevanten Veränderungen der Plasmaspiegel zu erwarten. Unter dem moderaten CYP2D6-Inhibitor Abirateron ist wiederum ein leichter Anstieg von Duloxetin, Fluoxetin und Venlafaxin nicht auszuschließen ( Tab. 12 ).

**Table:** **Tab. 12** Mögliche Auswirkungen verschiedener antiandrogen wirksamer Arzneimittel auf die Plasmakonzentrationen ausgewählter Antidepressiva aus der Gruppe der selektiven Serotoninwiederaufnahmehemmer (SSRI) und Serotonin/Noradrenalin-Wiederaufnahmehemmer (NaSRI) (mod. nach 3 21 28 64 72 ).

Antidepressiva (Abbauwege)	Abirateron	Apalutamid	Enzalutamid	Darolutamid
Citalopram (CYP2C19 > 3A)		↓↓↓	↓↓↓	
Desvenlafaxin (CYP2C19, UGT > CYP3A)		↓↓	↓↓	
Duloxetin (CYP1A2 > 2D6)	↑			
Escitalopram (CYP2C19 > 3A)		↓↓↓	↓↓↓	
Fluoxetin (CYP2D6 > 2C19)	↑ (QT)	↓	↓	(↓)
Fluvoxamin (CYP2D6)	↑ (QT)			
Milnacipran (–)				
Mirtazapin (CYP2D6, 3A > 1A2)	↑	↓↓	↓↓	(↓)
Paroxetin (CYP2D6)	↑			
Sertralin (CYP2B6, 2C19, 3A u.a.)		↓↓↓	↓↓↓	↓
Venlafaxin (CYP2D6,2C19 > 3A)	↑↑ (QT)	↓	↓	(↓)
Vortioxetin (CYP2D6 > 3A)	↑↑	↓↓	↓↓	(↓)
CYP=Cytochrom-P450; QT=QT-Zeit; WW=Wechselwirkungen; (–)=CYP- und PgP-unabhängige Clearance ↑: Folge einer schwachen Inhibition ; ↑↑: Folge einer mäßigen Inhibition; (↓): Folge einer minimalen Induktion; ↓: Folge einer schwachen Induktion; ↓↓: Folge einer mäßigen Induktion; ↓↓↓: Folge einer starken Induktion				

Beim Mirtazapin sind die CYP450-Isoenzyme CYP2D6 und CYP3A4 an der Biotransformation beteiligt 39 . Im Rahmen einer Kombination mit einem CYP2D6-Inhibitor kann es daher zu einem Anstieg, und mit einem CYP3A-Induktor zu einer Senkung der Plasmakonzentration kommen. Daher sollten Patienten, die Mirtazapin in Kombination mit den starken CYP3A-Induktoren Apalutamid oder Enzalutamid einnehmen, in den ersten 4–8 Wochen nach dem Start der ARI-Therapie genauer überwacht werden. Milnacipran ist hingegen kein CYP-Substrat 69 , sodass keine relevanten Veränderungen der Plasmaspiegel zu erwarten sind ( Tab. 12 ).

## Weitere Antidepressiva und Koanalgetika

Neben den SSRI und NaSRI spielen auch viele weitere Antidepressiva – allen voran die trizyklischen Antidepressiva (TCA) – eine wichtige Rolle in der Psychopharmakotherapie. An der Biotransformation sind meistens verschiedene CYP-Isoenzyme beteiligt, von CYP2C19 über CYP3A, 2D6, 2C9 bis CYP1A2 ( Tab. 13 ). Die zu erwartenden Veränderungen der Plasmaspiegel sind in Verbindung mit moderaten CYP2D6-Inhibitoren (z.B. Abirateron) bzw. moderat bis stark wirksamen CYP2C9/2C19/3A-Induktoren nur mit erheblichen Einschränkungen abzuschätzen. Interaktionsstudien mit dem Enzyminduktor Rifampicin geben zumindest teilweise Aufschluss darüber, welchen Effekt Apalutamid bzw. Enzalutamid auf TCAs haben könnten ( Tab. 13 ) 21 28 .

**Table:** **Tab. 13** Mögliche Auswirkungen verschiedener antiandrogen wirksamer Arzneimittel auf die Plasmakonzentrationen ausgewählter trizyklischer Antidepressiva (TCA), Benzodiazepine und sonstiger ZNS-wirksamer Pharmaka (mod. nach 21 28 ).

TCA, Benzodiazepine u.a. (Abbauwege)	Abirateron	Apalutamid	Enzalutamid	Darolutamid
Amitriptylin (CYP2C19, 2D6 > 3A, 1A2 und 2C9)	↑↑ (QT)	↓↓↓	↓↓↓	
Buspiron (CYP3A)		↓↓↓	↓↓↓	↓
Clomipramin (CYP2C19, 2D6, 1A2 > 3A)	(↑)	↓↓↓	↓↓↓	
Diazepam (CYP2C19 > 3A)		↓↓↓	↓↓↓	↓
Doxepin (CYP2C19 > 2D6, 2C9 u.a.)		↓↓↓	↓↓↓	
Gabapentin (–)				
Lorazepam (UGT2B15)				
Midazolam (CYP3A)		↓↓↓	↓↓↓	↓
Nortriptylin (CYP2D6)	↑↑ (QT)			
Opipramol (CYP2D6)	↑↑			
Pregabalin (–)				
Trimipramin (CYP2C19, 2D6)	↑	↓↓↓	↓↓↓	
Zopiclon (CYP3A)		↓↓↓	↓↓↓	↓
CYP=Cytochrom-P450; QT=QT-Zeit; TCA=trizyklischer Antidepressiva; UGT=UDP-Glucuronosyltransferase; WW=Wechselwirkungen; (–)=CYP- und PgP-unabhängige Clearance ↑: Folge einer schwachen Inhibition ; ↑↑: Folge einer mäßigen Inhibition; ↓: Folge einer schwachen Induktion; ↓↓: Folge einer mäßigen Induktion; ↓↓↓: Folge einer starken Induktion				

## Ausgewählte Tumortherapeutika

Eine klinisch-pharmakokinetische Studie, die die Interaktion von Enzalutamid mit Cabazitaxel untersuchte, kam zu dem Ergebnis, dass die AUC von Cabazitaxel um ca. 22% gesenkt wurde 40 . Da Docetaxel nicht nur ein CYP3A-, sondern auch ein PgP-Substrat ist, könnte unter Apalutamid der induktive Effekt noch stärker ausfallen als mit Enzalutamid ( Tab. 14 ).

**Table:** **Tab. 14** Mögliche Auswirkungen verschiedener antiandrogen wirksamer Arzneimittel auf die Plasmakonzentrationen ausgewählter Tumortherapeutika, die beim metastasierten Prostatakarzinom (mPC) eingesetzt werden (mod. nach 28 30 36 ).

Tumortherapeutikum (Abbauwege)	Abirateron	Apalutamid	Enzalutamid	Darolutamid
Cabazitaxel (CYP3A)		↓–↓↓	↓–↓↓	(↓)
Docetaxel (CYP3A, PgP)		↓↓	↓–↓↓	(↓)
Olaparib (CYP3A4, 3A5)		↓↓↓	↓↓↓	↓–↓↓
Relugolix (CYP3A4, 3A5, PgP > 2C8)		↓↓↓*	↓–↓↓	
CYP=Cytochrom-P450; PgP=P-Glycoprotein; WW=Wechselwirkungen ↓: Folge einer schwachen Induktion; ↓↓: Folge einer mäßigen Induktion; ↓↓↓: Folge einer starken Induktion *: Die Fachinformation sieht bei der unvermeidlichen Kombination mit einem CYP3A/PgP-Induktor eine Verdopplung der Relugolix-Dosis optional vor. Allerdings hat eine Phase-II-Studie vor Kurzem gezeigt, dass trotz Beibehaltung der Standarddosen (d.h. 120 mg Relugolix/240 mg Apalutamid) die Testosteronspiegel <50 ng/dl gehalten wurden 73 .				

Der PARP-Inhibitor Olaparib zählt ebenfalls zu den CYP3A-Substraten. Untersuchungen mit dem CYP3A/PgP-Induktor Rifampicin haben gezeigt, dass im Rahmen einer gemeinsamen Anwendung die AUC des Olaparibs um ca. 87% vermindert sein kann 67 . Eine gleichzeitige Gabe von Enzalutamid oder Apalutamid und Olaparib ist daher kritisch zu sehen. Im Vergleich zu Olaparib werden die PARP-Inhibitoren Niraparib und Talazoparib (PgP-Substrat) nicht über CYP-Isoenzyme verstoffwechselt 7 74 . Aufgrund der Enzalutamid-assoziierten PgP-Inhibition wird Talazoparib im Rahmen einer Kombination derzeit unter Studienbedingungen (TALAPRO-2) nur mit 0,5 mg/Tag und nicht mit 1 mg/Tag (Standarddosis) verabreicht 8 . Rucaparib wird vornehmlich über CYP2D6, und in geringerem Ausmaß über CYP1A2 und CYP3A4 metabolisiert 75 . Sie sind allerdings beide derzeit nicht für die Behandlung des mPC zugelassen.

Seit Kurzem steht auch der GnRH-Rezeptorantagonist Relugolix zur oralen Therapie des mHSPC international zur Verfügung. Der Wirkstoff wird zum einen über die CYP-Isoenzyme CYP3A und CYP2C8 abgebaut, zum anderen ist Relugolix ein Substrat der Effluxpumpe PgP. Wechselwirkungsstudien mit dem starken CYP3A/PgP-Induktor Rifampicin haben gezeigt, dass die gemeinsame Anwendung zu einer AUC-Senkung des Relugolix um ca. 55% führt 76 .

Da Apalutamid ebenfalls als potenter CYP3A/PgP-Induktor zu klassifizieren ist, würde man im Rahmen einer Kombination aus Apalutamid und Relugolix ebenfalls mit einer Abnahme der Plasmakonzentration des GnRH-Rezeptorantagonisten rechnen. Eine kürzlich veröffentlichte Phase-II-Studie zeigte allerdings, dass Relugolix und Apalutamid in Standarddosen gemeinsam verabreicht werden konnten, ohne dass es zu kritischen Erhöhungen der Testosteronspiegel kam 73 . Größer angelegte Studien werden deshalb dieser Fragestellung genauer nachgehen müssen, wie sicher die Kombination aus Relugolix mit Enzalutamid bzw. Apalutamid ist ( Tab. 14 ). Hingegen ist unter Darolutamid und Abi/P kein Einfluss auf die klinische Pharmakokinetik von Relugolix zu erwarten.

## Antiandrogene in Kombination mit CYP3A-Induktoren bzw. mit CYP2C8-Inhibitoren

In den vorherigen Abschnitten wurde erläutert, welchen Einfluss Antiandrogene auf häufig eingesetzte Arzneimittel haben können, mit einem besonderen Augenmerk auf das von den Antiandrogenen ausgehende Interaktionsrisiko. In diesem Zusammenhang ist darauf zu achten, dass im Falle einer CYP-Inhibition (z.B. Abirateron und CYP2D6) die Wechselwirkung nach dem Absetzen des Inhibitors relativ rasch nachlässt, da nach 4–5× Halbwertszeit kein Inhibitor mehr im Plasma nachweisbar ist ( Tab. 15 ). Beim Absetzen eines Induktors bleibt der induktive Effekt hingegen länger erhalten, da dabei nicht nur die 4–5× Halbwertszeit des Induktors ( Tab. 15 ), sondern auch die allmähliche Degradation der mehrfach gebildeten Enzyme über 10(–14) Tage zusätzlich zu berücksichtigen ist.

**Table:** **Tab. 15** Metabolische Abbauwege und Wechselwirkungsprofile der Antiandrogene und des CYP17-Inhibitors Abirateron (mod. nach 1 25 26 31 37 38 ).

INN	metabolisierende Enzyme	Halbwertszeit	Itraconazol (CYP3A-, PgP-Inhibition)	Rifampicin (CYP3A-, PgP-Induktion)	sonstige Anmerkungen
Abirateron	SULT2A1 > CYP3A	ca. 15–24 h	(↑) (+15%)	↓↓ (–55%)	Komedikation mit Rifampicin vermeiden
Apalutamid	CYP2C8 > 3A4	ca. 3 Tage	↑ (+50%)	↓–↓↓ (–34%)	keine Dosisanpassung mit Rifampicin
Enzalutamid	CYP2C8 > 3A4/5	ca. 5,8 Tage (8–9 Tage*)	↑ (+30%)	↓–↓↓ (–37%)	Gemfibrozil (CYP2C8-Inhibitor) führt zu 2,3-facher AUC-Erhöhung
Darolutamid	CYP3A4, UGT1A1, 1A9	ca. 20 Stunden	↑ (+70%)	↓↓ (–72%)	Komedikation mit Rifampicin vermeiden
AUC=Fläche unter der Kurve (Area Under the Curve, beschreibt die Wirkstoffexposition über die Zeit); CYP=Cytochrom-P450; INN=internationaler Freiname; PgP=P-Glycoprotein; SULT=Sulfotransferase; UGT=UDP-Glucuronosyltransferase * aktiver Metabolit des Enzalutamids					

Aber auch die Antiandrogene selbst können durch eine Komedikation mit CYP3A-Induktoren bzw. CYP2C8-Inhibitoren in ihrer Pharmakokinetik mehr oder weniger stark beeinflusst werden ( Tab. 15 ).

Potente CYP3A-Induktoren wie Rifampicin können die Exposition der Antiandrogene Abirateron bzw. Darolutamid um 45 bzw. 72% senken 11 22 , sodass eine gemeinsame Anwendung – wenn möglich – vermieden werden sollte.

Für den Abbau von Enzalutamid spielt das CYP-Isoenzym CYP2C8 eine wesentliche Rolle. Der Lipidsenker Gemfibrozil, ein potenter CYP2C8-Inhibitor, führt daher zu einer relevanten AUC-Erhöhung von Enzalutamid, sodass eine Dosisreduktion des Antiandrogens erfolgen muss 21 . Unter diesen Vorzeichen ist auch die Notwendigkeit der Dosisreduktion von Enzalutamid um 50% beim gleichzeitigen Einsatz von Clopidogrel zu verstehen, da der Metabolit Clopidogrel-acyl-β-D-glucuronid moderate CYP2C8-hemmende Eigenschaften aufweist 49 .

## Fiktiver Patientenfall zu relevanten Medikamenteninteraktionen in der Therapie des metastasierten Prostatakarzinoms

### Therapieverlauf

**Abb. 1:**
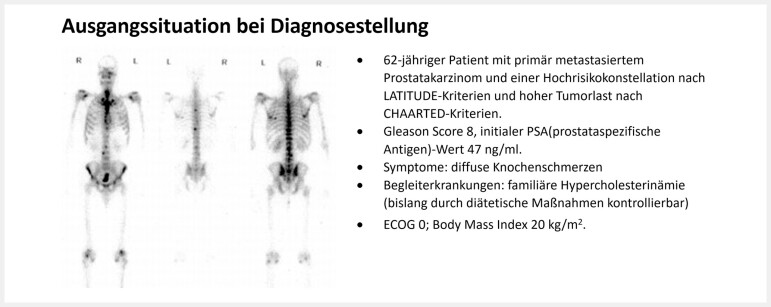
Ausgangssituation bei Diagnosestellung.

Etwa 6 Wochen nach Therapieeinleitung mit Leuprorelin und Apalutamid trat eine Besserung der Beschwerden ein, der PSA-Wert war deutlich rückläufig ( Abb. 1 ). Subjektiv war die Therapie sehr gut verträglich. Allerdings trat eine behandlungsbedürftige arterielle Hypertonie auf. Von seinem Hausarzt erhielt der Patient Amlodipin. Die Wirkung in der üblichen Dosierung war jedoch unzureichend. Ursächlich hierfür war vermutlich eine verminderte Plasmakonzentration durch eine ARPI-vermittelte CYP3A4-Induktion.

Nur wenige Wochen später erlitt der Patient einen Zentralarterienverschluss und wurde notfallmäßig neurologisch behandelt. Es wurde eine Therapie mit Clopidogrel, Acetylsalicylsäure und Simvastatin eingeleitet. Mit dieser Medikation wurde der Patient erneut am Zentrum vorstellig. Aufgrund der zu befürchtenden Medikamenteninteraktionen mit Apalutamid erfolgte eine Konsultation der Apotheke. Es wurde auf moderate Interaktionen mit Clopidogrel und Simvastatin hingewiesen. Da Clopidogrel verstärkt durch Apalutamid aktiviert wird (und damit ein erhöhtes Blutungsrisiko besteht), Clopidogrel aber auch gleichzeitig die Exposition des ARPIs erhöhen kann (und es so eine Verstärkung der Nebenwirkungen bewirken kann), erfolgte eine Umstellung auf Prasugrel. Aufgrund des zu befürchtenden geringeren Plasmaspiegels von Simvastatin durch CYP3A4-Induktion wurde dieses durch Pravastatin ersetzt. Mit beiden Modifikationen waren die neurologischen Kollegen einverstanden. Der weitere Krankheitsverlauf gestaltete sich komplikationslos.

Anmerkung: Der Patient wurde vor der Zulassung von Darolutamid für das metastasierte HSPC behandelt.

**Fazit**

Medikamenteninteraktionen müssen frühzeitig erkannt werden, um Schaden vom Patienten abzuwenden.

## Diskussion

Arzneimittelwechselwirkungen (DDI) auf der Basis einer Induktion oder Inhibition von Phase-I-Enzymen der Biotransformation – allen voran den CYP-Isoenzymen –, aber auch von Phase-II-Enzymen sowie von Influx- und Effluxtransportern, können zu erheblich veränderten Plasmaspiegeln in einer Komedikation führen. Dass dabei auch die Wirksamkeit der Komedikation deutlich abgesenkt werden kann, wird an dem dargestellten Fallbeispiel mit dem ARI Apalutamid und den CYP3A-Substraten Amlodipin und Simvastatin deutlich – sowohl die antihypertensive als auch die lipidsenkende Wirkung der beiden Arzneistoffe waren kaum mehr nachweisbar. Es ist nicht auszuschließen, dass die aktuellen Real-World-Daten zur Therapie mit Enzalutamid und Apalutamid beim nmCRPC, die von einem Gesamtrisiko von 40% für das Auftreten von Nebenwirkungen unter der Therapie, einer Abbruchquote von 10% und einer Hospitalisierungsrate von 5% teilweise mit DDI in Verbindung stehen könnten 33 . Auch für die häufig verordneten CYP2C19-Substrate Pantoprazol oder Clopidogrel besteht ein relevantes klinisch-pharmakokinetisches Interaktionsrisiko unter einer Therapie mit Apalutamid und Enzalutamid. Ob sich daraus auch ein erhöhtes Blutungs- und Hospitalisierungsrisiko ergibt, lässt sich aus den veränderten Plasmaspiegeln der Komedikationen nicht unmittelbar ableiten, sodass es am Ende immer auch klinischer Daten zur veränderten Wirksamkeit bzw. Toxizität bedarf, um die klinische Relevanz einer pharmakokinetisch basierten DDI tatsächlich zu erfassen 77 . In diesem Zusammenhang sei auch an das Beispiel mit Apalutamid und Relugolix erinnert: Unter Beibehaltung der Standarddosierungen ist eine Halbierung der Plasmaspiegel des oralen GnRH-Rezeptorantagonisten zu erwarten, woraus regulatorisch eine Verdoppelung der Relugolix-Dosis als Empfehlung abgeleitet wurde. Phase-II-Studienergebnisse konnten hingegen unter Beibehaltung der Standarddosen keine kritische Veränderung der Testosteronspiegel erkennen 73 ! Dieser Fall macht deutlich, dass weitergehende Studien zu dieser DDI noch mehr Klarheit schaffen müssen.

Selbst der endogene Stoffwechsel des Körpers kann durch starke CYP3A-Induktoren beeinträchtigt werden, wie Studien mit den CYP3A/2C-induzierenden Antiepileptika (z.B. Carbamazepin, Oxcarbazepim, Phenytoin, Phenobarbital) zeigen. Ihr kontinuierlicher Einsatz hatte die Vitamin-D-Biotransformation und -inaktivierung derart beschleunigt, dass die Patienten im Laufe der Therapie eine Hypovitaminose und eine damit verbundene, erhöhte Rate von Rachitis und Knochenbrüchen entwickelten 63 ! Eine engmaschige Kontrolle des Vitamin-D-Status ist in diesem Zusammenhang vor allem auch bei den CYP2C/3A-induzierenden ARI Apalutamid und Enzalutamid anzuraten, um frühzeitigen Knochenverlusten entgegenzuwirken 32 .

Weitere klinisch bedeutsame Bereiche, die eines differenzierten Komedikations-Managements bedürfen, stellen beispielsweise die analgetische Therapie bei symptomatisch metastasierten Patienten oder die Wahl des Antihypertensivums im Kontext einer ARI-Therapie dar.

Die Komplexität der pharmakologischen Interaktionspotenziale und ihrer Mechanismen ist hoch. Dies wird zusätzlich dadurch verstärkt, dass es beachtliche interindividuelle, z.T. populationsgenetische Variabilitäten (Polymorphismen) in der Expression von Cytochrom-P450-Enzymen, Efflux- und Influxpumpen gibt 78 79 .

Zahlreiche Datenbanken und Interaktionskalkulatoren bieten heute Hilfestellungen bei der Vorhersage möglicher Wechselwirkungen. Allerdings bedarf es immer wieder grundlegender Kenntnisse über die wirkstoffassoziierten Biotransformationswege, um eigenständig die Konsequenzen besser abschätzen zu können, die sich aus der Gabe eines Induktors bzw. Inhibitors im Rahmen einer Komedikation ergeben können. Dies ist am Ende wahrscheinlich zielführender, als sich ausschließlich auf eine Datenbank oder einen Interaktionsrechner zu verlassen, insbesondere wenn diese schwerpunktmäßig In-vitro-Ergebnisse zur Vorhersage möglicher Interaktionen berücksichtigen 4 5 6 7 . Nicht selten führt dann ein „Over-Alerting“ zu erheblichen Verunsicherungen beim behandelnden Arzt.

Die hier dargestellte Übersicht der Biotransformationswege unterschiedlicher Wirkstoffgruppen sowie die sich daraus ergebenden möglichen Effekte der verschiedenen ARI und Abirateron auf ihre klinische Pharmakokinetik können helfen, veränderte Wirkungen bzw. Nebenwirkungen im Rahmen einer Komedikation besser abschätzen und vermeiden zu können. Dadurch kann die Arzneimitteltherapiesicherheit in der täglichen Praxis erhöht werden. Allgemein fordert die zunehmende Komplexität der Medikamentenprofile und der, nicht zuletzt auch im Zeitalter der antitumoralen Kombinationstherapien, steigenden Relevanz von Interaktionsprofilen von Pharmaka, eine gesteigerte Aufmerksamkeit in der täglichen Praxis. Dies legt die Integration einer systematischen Analyse der Komedikation im Zuge interdisziplinärer Therapieentscheidungen, z.B. auch im Rahmen eines urologischen Tumorboards, nahe.
